# Early Patient Outcome and Left Ventricular Function After Mitral Valve Replacement Using a Tilting Disc Mechanical Valve With Total Preservation of Mitral Valvular Apparatus

**DOI:** 10.7759/cureus.74823

**Published:** 2024-11-30

**Authors:** Vikas Deep Goyal, Akhilesh Pahade, Amit Varshney

**Affiliations:** 1 Surgery, Shri Ram Murti Smarak Institute of Medical Sciences, Bareilly, IND; 2 Anesthesiology, Shri Ram Murti Smarak Institute of Medical Sciences, Bareilly, IND; 3 Internal Medicine, Shri Ram Murti Smarak Institute of Medical Sciences, Bareilly, IND

**Keywords:** mechanical tilting disc valve, mitral valve replacement, mitral valvular apparatus, total preservation, ttk chitra prosthetic valve

## Abstract

Introduction

The study aimed to retrospectively evaluate the early patient outcome and left ventricular function after mitral valve replacement with a tilting disc valve and total preservation.

Patients and methods

This retrospective observational study includes patients who underwent mitral valve replacement using a tilting disc valve with total preservation of mitral valvular and subvalvular apparatus from July 2021 to August 2022 at a single center.

Results

The data were reviewed retrospectively for age, sex, comorbidities, operating time, aortic cross-clamp time, cardiopulmonary bypass time, preoperative and postoperative left ventricular ejection fraction, mean gradient across the mitral valve, left ventricular diameter, left atrial size, atrial fibrillation, complications, mortality, and early patient outcome. Echocardiographic and clinical data were compared for the preoperative and postoperative periods. A significant difference in the preoperative and postoperative left atrial size, pulmonary arterial pressure, New York Heart Association class, and mean gradient across the mitral valve was found. However, the difference between the preoperative and postoperative ejection fraction and left ventricular diameter in systole and diastole was not significant.

Conclusion

Early patient outcome after mitral valve replacement with a tilting disc valve and total preservation of mitral valvular apparatus gives good results with minimal morbidity and mortality. Favorable patient outcomes in this study lay to rest any skepticism regarding total preservation while using a tilting disc valve. The technique of total preservation is safe while using a tilting disc valve, and it helps in the preservation of the left ventricular ejection fraction and gives a low gradient across the valve.

## Introduction

Rheumatic heart disease (RHD) is common in developing countries and is a major cause of valvular heart disease, whereas myxomatous and degenerative valvular diseases are common in the Western world [1]. In RHD, the mitral valve is the most commonly affected valve, followed by the aortic valve. Options in the management of such patients, apart from medical treatment, include valvotomy for stenotic lesions, mitral valve repair for regurgitant lesions, and mitral valve replacement (MVR) for severely thickened, damaged, and calcified valves not suitable for repair. Either mechanical or bio-prosthetic valves are used during MVR [1,2]. In mechanical valves, bileaflet valves are more commonly used than a single leaflet or tilting disc valve, although both types of valves give good results [2,3]. The lower cost of tilting disc valves helps reduce the overall cost of the procedure, especially in developing countries [3]. The techniques of MVR include the classical technique, where the whole of the mitral valve is excised; the posterior mitral leaflet (PML) preserving technique, where PML is preserved, and anterior mitral leaflet (AML) is removed with chordae tendineae; and the technique of total preservation where both the AML and PML are preserved [4,5]. There are studies available in the literature comparing classical MVR with the preservation of PML, as well as studies comparing the preservation of PML with total preservation [4,5]. The majority of the studies on total preservation have been done using bileaflet mechanical valves, with few studies available on total preservation using tilting disc valves [5,6]. In the case of tilting disc valves, most of the studies are done with the preservation of PML only. The literature on tilting disc valves along with total preservation of mitral valvular apparatus is limited.

This study aimed to retrospectively evaluate the early patient outcome and left ventricular (LV) function after MVR using a tilting disc valve (TTK Chitra, TTK Healthcare, Chennai, India), along with total preservation of the mitral valvular apparatus. Our study adds to the limited literature on tilting disc valves and total preservation.

## Materials and methods

Study design

This retrospective observational study includes patients who underwent MVR using tilting disc valves along with total preservation from July 2021 to August 2022 at a single center.

Data collection

Patient-related variables were collected from the hospital database, and follow-up was done both from hospital records and by telephone.

Inclusion/exclusion criteria

Patients who underwent MVR using the total preservation technique were evaluated during the study period. Patients who underwent MVR with a tilting disc valve were included in the study. Patients who underwent double valve replacement (DVR) or other simultaneous cardiac procedures were not included in the study. Patients with an ejection fraction of less than 35% were not included in the study.

Statistical analysis

The statistical tests that were applied to the data are as follows. 1) Mean and standard deviation were used for the scale variables. 2) For the categorical variables, frequency and percentages were used. 3) For the two-variable analysis, a paired t-test was used where both the variables measured were scale variables, and since the two variables were compared for the same patient. 4) For the two variables where both the variables were measured as categorical, cross-tabulation between the variables was conducted, and to draw conclusions based on statistical inferences, the chi-square test was used. The analysis was conducted with the help of SPSS (IBM SPSS Statistics for Windows, IBM Corp., Version 20, Armonk, NY) software.

Operative technique

**Figure 1:**
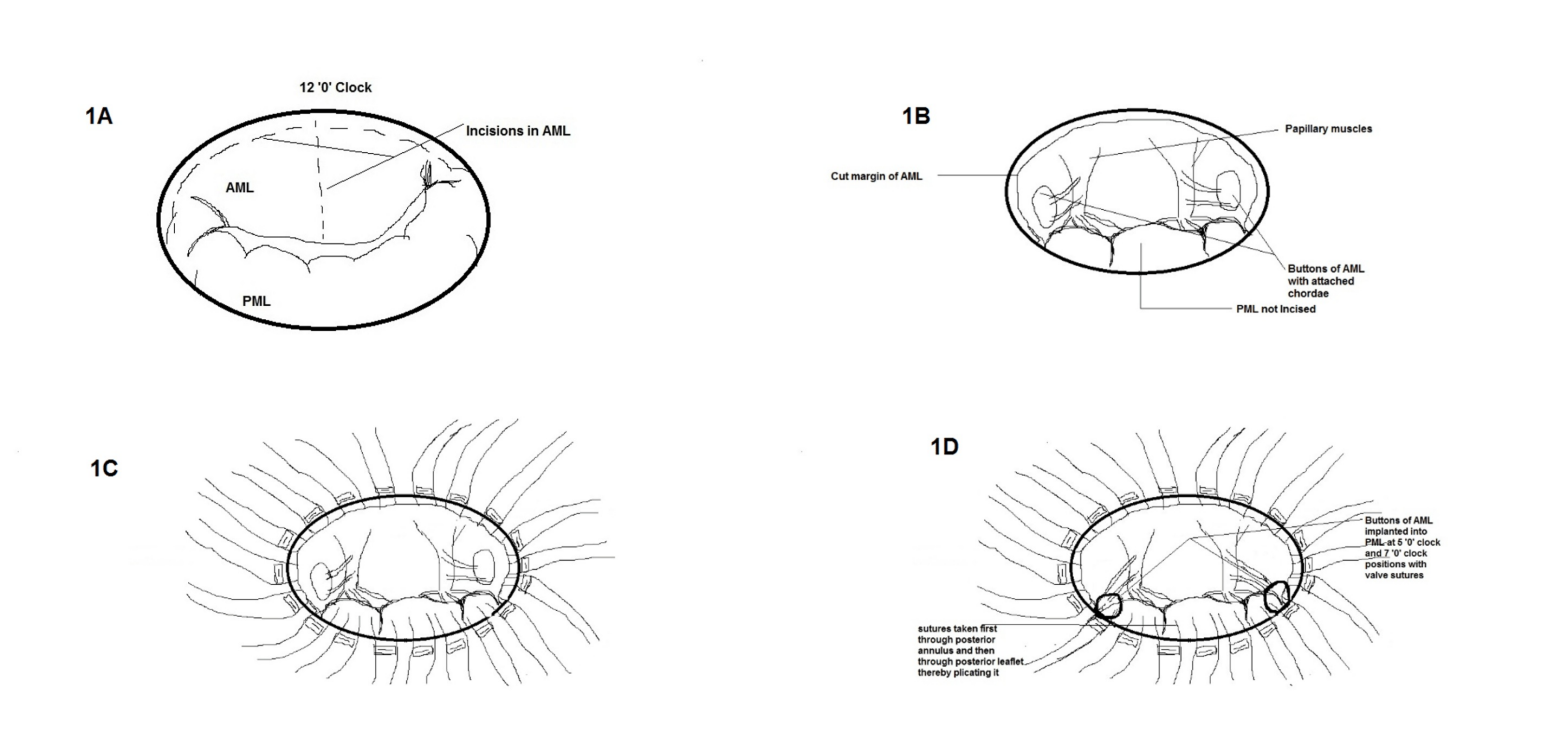
Diagrammatic illustration of total preservation technique. 1A - Incisions in AML. 1B - Creation of buttons of AML. 1C - Pledgeted everting sutures taken in mitral annulus. 1D - Buttons of AML implanted into PML at 5 and 7 o’clock positions. AML - anterior mitral leaflet, PML - posterior mitral leaflet Source: Image created by the first author on 11/02/2023.

**Figure 2:**
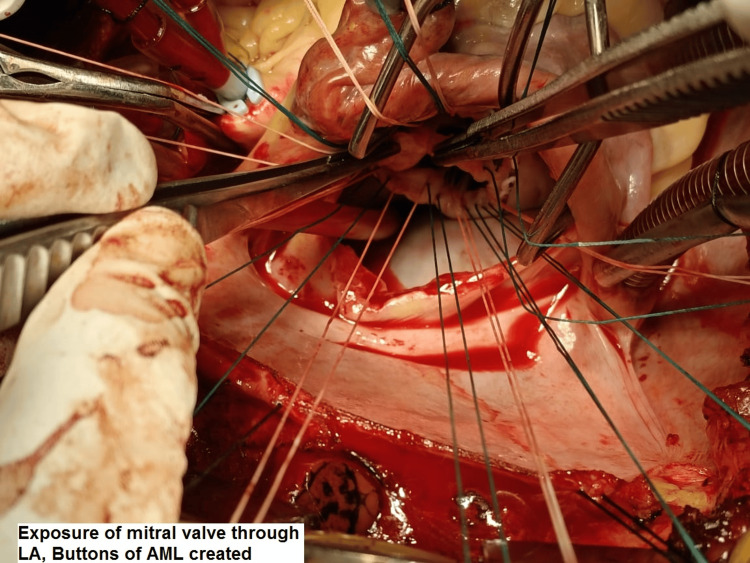
Anterior mitral leaflet buttons after trimming of redundant leaflet tissue for re-implantation into the posterior annulus.

**Figure 3:**
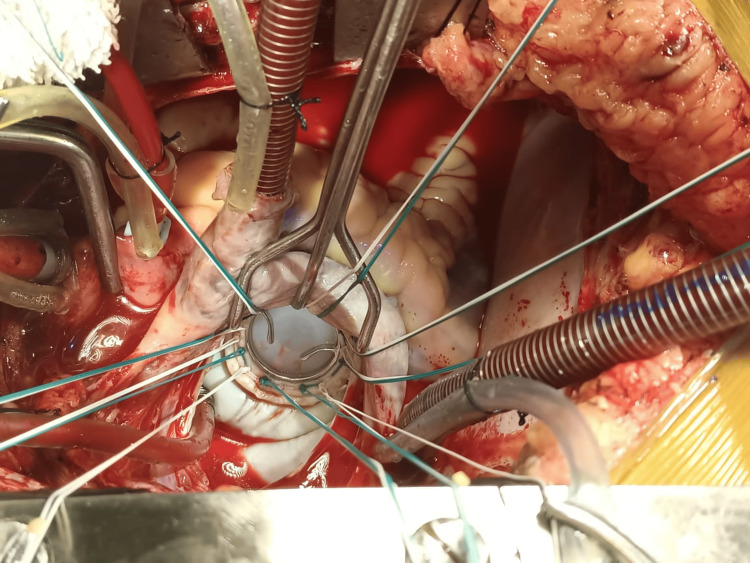
Implanted TTK Chitra tilting disc valve in closed position.

**Figure 4:**
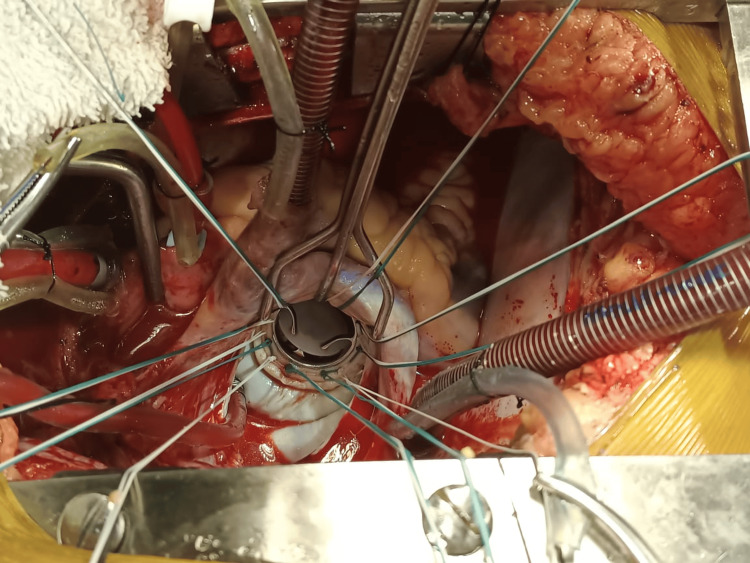
Implanted TTK Chitra tilting disc valve in an open position, major orifice is anteriorly placed.

All the surgeries were performed by the same surgical team. Median sternotomy was the surgical approach. Cannulation for cardiopulmonary bypass (CPB) was done after heparinization (4 mg/kg). Ascending aortic and bicaval cannulation (through the right atrium) was done. Antegrade blood cardioplegia was used. The mitral valve was approached through the left atrial approach behind Sondergaard's plane. The left atrium (LA) and appendage were inspected for any clots, and clots were removed if present. The mitral valve was then assessed, the AML was divided into two halves, excess leaflet tissue along with calcifications, if found, were removed, and two buttons of the leaflet with attached chordate were created (Figures 1A-1B). PML and chordae were kept intact unless there were calcifications that were removed with rongeur forceps. Pledgeted 2-0 polyester sutures (Trubond, Healthium Medtech Limited, Bengaluru, India) were taken in the mitral annulus in an everting technique (Figure 2). Sutures in the PML were taken first from the annulus and again through the leaflet edges, sandwiching the leaflet tissue. The two buttons of the AML were transposed to the PML and fixed with valve sutures mostly in 4-5 o’clock and 7-8 o’clock positions, respectively (Figures 1C-1D). The appropriate valve was then selected by the valve sizer, and the TTK Chitra mechanical valve was then implanted using the previously placed valve sutures, keeping the position of the major orifice of the prosthetic valve anteriorly (Figure 3 and Figure 4). The LA was then closed using polypropylene sutures 4-0. The left atrial appendage was ligated in patients who were in atrial fibrillation (AF) rhythm or if there was a presence of LAA clot from outside using no. 1 silk suture. Standard de-airing techniques were followed before coming off bypass.

Postoperative care and follow-up

The patients were kept in the intensive care unit for two days. Patients were ventilated overnight and were extubated the next morning. Echocardiography was done on the first postoperative day as well as at the time of discharge. Most patients were discharged on the sixth postoperative day and were called for follow-up after one week. Local examination of the wound and clinical assessment of valve click was also done at that time. The first follow-up was after one week, then after one month, and thereafter every three months for the first year. Prothrombin time (PT)/international normalized ratio (INR) was checked at each follow-up, and INR was maintained between two and 2.5 for patients in sinus rhythm and 2.5 and three for patients in AF. Standard instructions were given for dietary and general care. Echocardiography was done at six monthly follow-ups and thereafter annually.

## Results

Twenty-two patients underwent MVR using a tilting disc (TTK Chitra) mitral mechanical valve with total preservation of mitral valvular apparatus over one year from July 2021 to August 2022. The data were reviewed retrospectively for age, sex, comorbidities, operating time, aortic cross-clamp (ACC) time, CPB time, preoperative and postoperative LV ejection fraction, LV diameter, left atrial size, AF, mean gradient across the mitral valve, complications, and mortality (Table 1). The mean age of patients in the study was 34.2 ± 9.76 years. The male-to-female ratio in the study was 1:1. The predominant lesion was RHD with severe mitral stenosis (MS) with moderate to severe mitral regurgitation (MR) in 14 (63.63%) patients. The mean ACC time was 86.68 ± 15.56 minutes, and the mean CPB time was 113.95 ± 18.67 minutes. The mean operating time was 5.82 ± 0.92 hours. The most common implanted valve was of 25 mm size in eight (36.36%) patients, followed by a 27 mm valve in six (27.27%) and a 29 mm valve in five (22.72%) patients. Two patients were implanted with 31 mm size valves, and in one patient, 23 mm size valves were implanted. Operative survival was 100%. Echocardiographic parameters like LV function, mean diastolic gradient (MDG), LA size, systolic pulmonary artery pressure (SPAP), LV end-diastolic diameter (LVEDD), and LV end-systolic diameter (LVESD) were compared between preoperative and postoperative values (Table 2). There was a significant difference in the SPAP, MDG, and LA size between preoperative and postoperative values, whereas for LV ejection fraction, LVESD, and LVEDD, the difference was insignificant. Clinical parameters like the New York Heart Association (NYHA) class and AF were also compared for preoperative and postoperative values, and a significant difference was found (Table 2). Two patients required re-exploration for bleeding. One patient had a wound infection, which prolonged the hospital stay. There was no operative mortality. Follow-up was completed for 21 (95.45%) patients; one patient did not come for follow-up after five months post-procedure. The mean follow-up time was 10.15 ± 5.42 months. During the follow-up period, one patient had a pericardial collection one-month post-procedure, which required drainage using a pigtail catheter under echocardiographic guidance. The patient was discharged after five days. Another patient had intra-abdominal bleeding 15 months post-procedure due to high INR, and the diagnosis of a ruptured corpus luteum cyst was made. The patient was treated conservatively with fresh frozen plasma, blood transfusion, and other conservative measures and was discharged after seven days. All other patients were doing well, and none of the patients had a mean gradient across the mitral valve of more than 5 mmHg on repeat echocardiography at six months.

**Table 1:** Combined demographic and clinical data of patients in the study. Values expressed as count (percent) or mean ± standard deviation. AF - atrial fibrillation, ACC - aortic cross-clamp, CPB - cardiopulmonary bypass, EF - ejection fraction, HS - hospital stay, LA - left atrium, LAA - left atrial appendage, MR - mitral regurgitation, MS - mitral stenosis, RHD - rheumatic heart disease, OT - operating time, (++) - moderate severity

Demographic and clinical parameters	Total sample
Demographic parameters	
Age (in years)	34.2 ± 9.76
Male sex (%)	11(50)
Clinical parameters	
RHD severe MS (++MR)	14 (63.63%)
RHD severe MR (++MS)	8 (36.67%)
HS (days)	9.5 ± 1.65
OT (hours)	5.82 ± 0.92
ACC time (minutes)	86.68 ± 15.56
CPB time (minutes)	113.95 ± 18.67
LAA clot (%)	7 (32)
AF rhythm (%)	14 (63.6)
LAA ligation (%)	20 (90.9)
Complications	
Pericardial collection (%)	1 (4.5)
Re-exploration (%)	2 (9.1)
Wound infection (%)	1 (4.5)
Mortality	0 (0)

**Table 2:** Comparison of preoperative and postoperative echocardiographic and clinical data. AF - atrial fibrillation, EF - ejection fraction, LA - left atrium, LVEDD - left ventricular end-diastolic diameter, LVESD - left ventricular end-systolic diameter, NYHA - New York Heart Association, SPAP - systolic pulmonary artery pressure Values expressed as mean ± standard deviation or absolute numbers, *** significant at 95% levels.

S. no.	Echocardiographic and clinical parameters	Mean ± SD/number of patients	p-value
1	Preoperative EF (%)	54.32 ± 6.23	0.541
	Postoperative EF (%)	53.18 ± 7.49	
2	Mean preoperative gradient	13.95 ± 3.29	0***
	Mean postoperative gradient	3.32 ± 0.99	
3	Preoperative NYHA class	2.95 ± 0.65	0***
	Postoperative NYHA class	1.5 ± 0.60	
4	Preoperative LA size (mm)	63.95 ± 11.36	0***
	Postoperative LA size (mm)	53.45 ± 8.11	
5	Preoperative SPAP (mmHg)	68.27 ± 9.87	0***
	Postoperative SPAP(mmHg)	45.55 ± 9.65	
6	Preoperative LVEDD (mm)	50.32 ± 3.67	0.083
	Postoperative LVEDD (mm)	49.64 ± 3.61	
7	Preoperative LVESD (mm)	31.5 ± 3.11	0.687
	Postoperative LVESD (mm)	31.68 ± 3.62	
8	Preoperative AF	14	0.022***
	Postoperative AF	07	

## Discussion

Results in this study on tilting disc valves with total preservation of mitral valvular apparatus were consistent with improved patient outcome, preserved LV function, and lesser incidence of postoperative arrhythmia. A slight prolongation of CPB time and ACC time was also noted in the study. The tilting disc valve used in this study was TTK Chitra. The TTK Chitra heart valve was developed by TTK Healthcare (India). The valve has been in use for more than three decades. The results of the TTK Chitra valve are comparable to other mechanical valves being used [7,8]. The safety and efficacy of the valve have been proved in several midterm and long-term studies [7,8]. The lower cost of the tilting disc valve in developing countries is an added advantage [7,8]. The cost of the tilting disc valve used in this study in our country (India) is approximately $450 (US dollar), and that of bileaflet valves is above $700. This difference may not be large for developed countries but is significant for developing countries.

Various techniques of total preservation have been reported in the literature. One of the commonly used techniques of total preservation is splitting the AML into two halves and creating two buttons that are either fixed on the posterior leaflet or fixed near the commissures [9]. With tilting disc valves fixing the buttons of the anterior leaflet posteriorly gives more space anteriorly for the free movement of the valve disc. Some technical points might be helpful while implanting tilting disc valves. 1) Apart from annulus size, subjective assessment of LV cavity size is important in choosing an appropriate-sized valve. 2) Avoid implanting oversized valves, especially with small LV cavities. 3) Avoid using oversized valves in the stenotic calcified annulus, as the annulus is not stretchable. 4) Shaving of the PML in stenotic calcified valves with number 15 surgical blade. 5) Shaving of thickened chordae of AML in cases with excessively thickened chordae. 6) The major orifice of the valve is kept anteriorly. In the case of bileaflet valves, the buttons of the anterior leaflet may be fixed near the commissures or sometimes left at the site of the native anterior leaflet. Our preference for total preservation with a tilting disc valve is shifting the buttons of the AML posteriorly, and for bileaflet valves is shifting the buttons of the AML laterally near the commissures. There are reports on the preservation of the mitral valve at the native position in patients with a large LA with good results [10].

Advantages of total preservation include decreased chances of LV dysfunction, better hemodynamic performance, a lesser requirement of inotropes, better patient outcome, and more chances of preserved LV function over the long term. These advantages were also seen in this study while using tilting disc valves. Most of the studies on total preservation have been done using bileaflet valves. A comparative study on MVR in patients of mitral incompetence with total versus partial chordal preservation using St. Jude Medical bileaflet mechanical prosthesis (St. Jude Medical, Saint Paul, MN) concluded that total preservation is associated with more favorable patient outcomes in terms of morbidity, mortality, and LV function [11]. Factors that make total preservation difficult include densely calcified valves, severe sub-valvular crowding, small-sized LV cavities, and the presence of infective endocarditis or valvular abscess.

Total preservation may be associated with a prolongation of the ACC and CPB time, which was also seen in this study. Total preservation with a tilting disc valve may require some experience with its use. Trimming of the anterior leaflet buttons before re-implantation and splitting incisions in the posterior leaflet, along with debridement of calcium, is generally helpful in minimizing the chances of implantation of an undersized valve. ACC time and CPB time were on the higher side in our study, also due to the time spent creating the appropriate size buttons and locating a suitable site for their fixation to avoid impingement on the valve disc. A comparative study on total versus partial preservation concluded that total preservation is associated with lesser morbidity and mortality and better LV function, although with higher ACC time and CPB time [12]. Another study in the literature reported longer ACC time using the technique of total preservation as compared to the preservation of PML only or classical technique. This study also reported improved LV function after total preservation [13]. Mitral valve repair has also been compared with MVR using total preservation, and similar results were found with both techniques [14]. A comparison of total preservation with PML preservation and classical technique revealed better LV volume and function in the total preservation group, although ACC time was longer [15]. Lafci et al. used the technique of papillary muscle repositioning [16]. Preservation of PML was their standard technique using bileaflet valves. In cases of RHD with MS in which the posterior leaflet could not be preserved due to fibrosis or calcification, they used the papillary muscle repositioning technique and reported good results.

Sá et al. did a meta-analysis of 20 studies in the literature and reported a significantly lower incidence of operative mortality, low output syndrome, and five-year mortality after MVR with preservation of subvalvular apparatus in comparison to those without preservation of subvalvular apparatus [17]. In another meta-analysis by Hsieh et al., there was a significant benefit in long-term survival in cases with MVR with preservation rather than without preservation, but the short-term benefit was not found [18]. These two meta-analyses, however, differed in the early results of MVR with preservation from that without preservation, which may need to be further analyzed. Although our study included a smaller number of patients, the early and short-term results with the total preservation technique were analyzed, and they were highly rewarding.

The use of tilting disc valves has decreased in recent times despite good patient outcomes. There is skepticism regarding disc movement while doing total preservation using tilting disc valves, and PML preservation is mostly done along with these valves. We observed that by shifting the AML to the posterior position, a lot of space is created anteriorly for the free motion of the disc. We did not need to reimplant any valve in this series for disc obstruction. This study helps to remove any skepticism regarding total preservation while using tilting disc valves. Minimal impairment of LV function in the postoperative period with total preservation more than compensates for the slightly prolonged CPB and ACC time.

Although we used the standard median sternotomy approach in our patients, minimally invasive techniques using partial sternotomy and right thoracotomy have been reported to give good results [19]. Even robotic-assisted valve procedures are being done at some centers, and the role of technology is bound to increase [20].

Limitations of the study

A smaller number of patients and a lack of a comparison group were the drawbacks of this study. The study evaluated early and short-term results but not midterm and long-term results.

## Conclusions

Total preservation of mitral valvular and subvalvular apparatus while using a tilting disc valve for MVR is a safe and effective technique and preserves LV function. Favorable patient outcomes in this study lay to rest any skepticism regarding total preservation while using a tilting disc valve. The mean gradient after valve replacement also comes within the normal range. Early patient outcomes using this technique were favorable, with minimal morbidity and mortality.

## References

[1] Yu J, Wang W (2022). Bioprosthetic vs. mechanical mitral valve replacement for rheumatic heart disease in patients aged 50-70 years. Front Cardiovasc Med.

[2] Malhotra A, Pawar SR, Srivastava A, Yadav BS, Kaushal R, Sharma P, Songra M (2014). Clinical and hemodynamic study of tilting disc heart valve: single-center study. Asian Cardiovasc Thorac Ann.

[3] Kaushik R, Mani A, Ganapathi S, Pillai VV, Jayakumar K, S H (2022). Clinical outcomes of bileaflet St. Jude Medical and tilting disc TTK Chitra mechanical heart valve prosthesis: a comparative study. J Card Surg.

[4] Borger MA, Yau TM, Rao V (2002). Reoperative mitral valve replacement: importance of preservation of the subvalvular apparatus. Ann Thorac Surg.

[5] Ucak A, Ugur M, Onan B, Arslan G, Alp I, Ulusoy E, Yilmaz AT (2011). Conventional versus complete chordal-sparing mitral valve replacement: effects on left ventricular function and end-systolic stress. Acta Cardiol.

[6] Feikes HL, Daugharthy JB, Perry JE, Bell JH, Hieb RE, Johnson GH (1990). Preservation of all chordae tendineae and papillary muscle during mitral valve replacement with a tilting disc valve. J Card Surg.

[7] Sankarkumar R, Bhuvaneshwar GS, Magotra R (2001). Chitra heart valve: results of a multicenter clinical study. J Heart Valve Dis.

[8] Varma PK, Vijayakumar M, Bhuvaneshwar GS, Kumar AS, Krishna N (2023). Long-term evaluation of TTK Chitra™ heart valve prosthesis - a retrospective-prospective cohort study. Indian J Thorac Cardiovasc Surg.

[9] Ozdemir AC, Emrecan B, Baltalarli A (2014). Bileaflet versus posterior-leaflet-only preservation in mitral valve replacement. Tex Heart Inst J.

[10] Chen L, Chen B, Hao J (2013). Complete preservation of the mitral valve apparatus during mitral valve replacement for rheumatic mitral regurgitation in patients with an enlarged left ventricular chamber. Heart Surg Forum.

[11] Hassouna A, Elmahalawy N (1998). Valve replacement in rheumatic mitral incompetence: total versus posterior chordal preservation. Cardiovasc Surg.

[12] Cingöz F, Günay C, Kuralay E (2004). Both leaflet preservation during mitral valve replacement: modified anterior leaflet preservation technique. J Card Surg.

[13] Chen SX, Xi EP, Zhang WX (2003). Preservation of the entire mitral subvalvular apparatus during mitral valve replacement in patients with mitral stenosis. Hunan Yi Ke Da Xue Xue Bao.

[14] Kisamori E, Otani S, Yamamoto T, Nishiki M, Yamada Y, Matsumoto T (2019). Mitral valve repair versus replacement with preservation of the entire subvalvular apparatus. Gen Thorac Cardiovasc Surg.

[15] Guo Y, He S, Wang T, Chen Z, Shu Y (2019). Comparison of modified total leaflet preservation, posterior leaflet preservation, and no leaflet preservation techniques in mitral valve replacement - a retrospective study. J Cardiothorac Surg.

[16] Lafci G, Cagli K, Cicek OF (2014). Papillary muscle repositioning as a subvalvular apparatus preservation technique in mitral stenosis patients with normal left ventricular systolic function. Tex Heart Inst J.

[17] Sá MP, Ferraz PE, Escobar RR (2012). Preservation versus non-preservation of mitral valve apparatus during mitral valve replacement: a meta-analysis of 3835 patients. Interact Cardiovasc Thorac Surg.

[18] Hsieh WC, Aboud A, Henry BM, Kan CD, Omara M, Lindner J, Kolesová H (2020). Mitral valve replacement using subvalvular apparatus: a systematic review and meta-analysis. Heart Surg Forum.

[19] Feirer N, Kornyeva A, Lang M (2022). Non-robotic minimally invasive mitral valve repair: a 20-year single-centre experience. Eur J Cardiothorac Surg.

[20] Güllü AÜ, Şenay Ş, Koçyiğit M, Ökten EM, Dumantepe M, Karabulut H, Alhan C (2019). The feasibility of robotic-assisted concomitant procedures during mitral valve operations. Turk Gogus Kalp Damar Cerrahisi Derg.

